# Personalized medicine: Function of CFTR variant p.Arg334Trp is rescued by currently available CFTR modulators

**DOI:** 10.3389/fmolb.2023.1155705

**Published:** 2023-03-17

**Authors:** Violeta Railean, Cláudia S. Rodrigues, Sofia S. Ramalho, Iris A. L. Silva, Jan Bartosch, Carlos M. Farinha, Ines Pankonien, Margarida D. Amaral

**Affiliations:** BioISI—Biosystems and Integrative Sciences Institute, Faculty of Sciences, University of Lisboa, Lisboa, Portugal

**Keywords:** cystic fibrosis, R334W, theranostics, personalized medicine, theratyping, organoids

## Abstract

Most of the 2,100 CFTR gene variants reported to date are still unknown in terms of their disease liability in Cystic Fibrosis (CF) and their molecular and cellular mechanism that leads to CFTR dysfunction. Since some rare variants may respond to currently approved modulators, characterizing their defect and response to these drugs is essential for effective treatment of people with CF (pwCF) not eligible for the current treatment. Here, we assessed how the rare variant, p.Arg334Trp, impacts on CFTR traffic and function and its response to existing CFTR modulators. To this end, we performed the forskolin-induced swelling (FIS) assay on intestinal organoids from 10 pwCF bearing the p.Arg334Trp variant in one or both alleles of the CFTR gene. In parallel, a novel p.Arg334Trp-CFTR expressing CFBE cell line was generated to characterize the variant individually. Results show that p.Arg334Trp-CFTR does not significantly affect the plasma membrane traffic of CFTR and evidences residual CFTR function. This CFTR variant is rescued by currently available CFTR modulators independently of the variant in the second allele. The study, predicting clinical benefit for CFTR modulators in pwCF with at least one p.Arg334Trp variant, demonstrates the high potential of personalized medicine through theranostics to extend the label of approved drugs for pwCF carrying rare CFTR variants. We recommend that this personalized approach should be considered for drug reimbursement policies by health insurance systems/national health services.

## 1 Introduction

Cystic Fibrosis (CF) is the most common life-shortening autosomal recessive disorder among Caucasians, estimated to affect ∼52,000 individuals in Europe and ∼105,000 individuals worldwide (ECFS, 2020; Guo et al., 2022). CF is caused by variants in the gene encoding the CF transmembrane conductance regulator (CFTR) protein, a cAMP-regulated chloride (Cl^−^) and bicarbonate (HCO_3_^−^) channel expressed at the apical plasma membrane (PM) of epithelial cells (Riordan et al., 1989; Sheppard and Welsh, 1999). Defective CFTR function leads to abnormal ion transport causing dehydration of the mucus lining of mucosal surfaces, among those, the lung and the intestine (Rowe et al., 2005).

To date, about 2,100 alterations have been described in the CFTR gene (CFMDB, 2021), but the disease liability is only established for less than a quarter of these (CFTR, 2023). These variants have been grouped into seven functional classes according to their molecular/cellular defect and these are now evolving into theratypes (De Boeck and Amaral, 2016). Indeed, the underlying concept is that variants within the same class are rescued by the same therapeutic strategy, if not by the same drug (Cutting, 2015). Although this classification in theratypes is helpful, the significance of most variants is still unknown, not only regarding disease liability, but also the respective underlying molecular/cellular defect (De Boeck and Amaral, 2016; Sabusap et al., 2016). The most common CFTR variant is the deletion of three base pairs that result in the loss of phenylalanine at position 508: p.Phe508del (legacy name: F508del) (De Boeck and Amaral, 2016; CFMDB, 2023; CFTR, 2023). This variant causes CFTR misfolding leading to retention in the endoplasmic reticulum (ER) by the ER quality control (ERQC), premature degradation and failure to reach the PM (Ramalho et al., 2021). In addition, the p.Phe508del variant has also been shown to have a gating defect characterized by a reduction in open probability (Po) resulting in an abnormal Cl^−^ transport (Dalemans et al., 1991). Identification of small molecules that rescue the p.Phe508del-CFTR folding/trafficking/gating defect lead to the approval of three different corrector drugs currently available for individuals with CF bearing this mutation in combination with ivacaftor (iva), a CFTR gating potentiator: lumacaftor (luma), tezacaftor (teza) or elexacaftor (elexa, combined with teza) (Van Goor et al., 2006; Jih and Hwang, 2013; Wainwright et al., 2015; Taylor-Cousar et al., 2017; Middleton et al., 2019). These drugs are variant-specific and accordingly, they have been developed for pwCF homozygous for p.Phe508del (luma/iva; teza/iva; and elexa/teza/iva) or bearing at least one p.Phe508del (elexa/teza/iva). Consequently, most pwCF who have rare variants are not eligible for these treatments, except for those with gating mutations (class III) which are likely to be responsive to the potentiator (iva) alone.

However, some of the non-eligible rare variants are likely to respond to approved CFTR modulators (De Boeck and Amaral, 2016; Sabusap et al., 2016; Ramalho et al., 2021). Moreover, it is also often found that pwCF with the same CFTR genotype have significantly different clinical responses to CFTR-modulating drugs (Awatade et al., 2015; Wainwright et al., 2015). There is thus an unmet need to test these novel CFTR modulators directly *ex vivo* on the individual’s tissues and/or primary cells with robust assays assessing modulator efficacy for a given CFTR genotype and thus predict clinical benefit for the donor individual.

One such rare variant, which is not included in the list of eligible variants for current CFTR modulators, is p.Arg334Trp. This is a missense variant which is located in transmembrane segment 6 (TM6) in the membrane spanning domain (MSD) one and characterized by decreased CFTR channel function not affecting channel folding or trafficking (Sheppard et al., 1993; Han et al., 2018). Previous studies have shown that the impairment is most likely caused by decreased conductance rather than Po since the treatment of p.Arg334Trp-CFTR expressing CFBE cells with potentiator iva only partly/slightly corrected its gating defect, i.e., to 5% of wt-CFTR transport activity (Veit et al., 2020). As such, p.Arg334Trp is included in class IV of CFTR variants (Sheppard et al., 1993). Moreover, in a different study it has also been observed that p.Arg334Trp-CFTR retained significant apical membrane Cl^−^ channel function. Clinically, individuals carrying p.Arg334Trp-CFTR are more likely to be pancreatic sufficient and often present atypical CF disease (CFTR, 2023).

Here, we focus on determining the effect of the rare p.Arg334Trp variant on CFTR traffic (processing) and function and its response to existing CFTR modulators in more physiological relevant cell models. To this end, we used intestinal organoids from 10 individuals carrying the p.Arg334Trp variant in one or both alleles of the CFTR gene. In parallel, we generated a human CF bronchial epithelial cell line (CFBE41o^−^) expressing CFTR with this variant in order to study this variant separately/independently from other variants.

## 2 Materials and methods

### 2.1 Chemicals and compounds

All chemicals were of analytical grade. DMSO, Forskolin and Genistein were obtained from Sigma Aldrich, ivacaftor, tezacaftor and elexacaftor from Selleckchem (United States), and CFTR-inh172 from MedChemexpress (United States). All compounds were dissolved in DMSO.

### 2.2 CF subjects and ethical approval

Rectal biopsies were obtained from 10 Portuguese individuals with CF carrying the p.Arg334Trp CFTR variant. One of these was homozygous for p.Arg334Trp and the other nine had the following variants in their second CFTR allele: p.Arg1162X, p.Gly542X, p.Tyr1092X, p.Phe508del (4 individuals), p.Gln1100Pro, p.Thr1086Pro (c.3256A>C). Organoids from one Portuguese individual with the p.Phe508del/p.Phe508del genotype were used as control. Informed consent was obtained from all the subjects and the study was conducted according to the guidelines of the Declaration of Helsinki and approved by the Ethics Committee of Hospital de Santa Maria, Lisboa, Portugal (DIRCLN-16JUL 2014–211). The individuals mean age was 34 years (interval: 26–46 years).

### 2.3 Human intestinal organoids and forskolin-induced swelling (FIS) assay

Crypt isolation from rectal biopsies, organoids culture and FIS assay were performed as described by Vonk et al. (Vonk et al., 2020). Organoids were treated with 0.02; 0.128; 0.8; and 5 μM of forskolin (fsk) alone or together with: only 3.33 μM iva; 5 μM teza +3.33 μM iva; or 3 μM elexa + 5 μM teza +3.33 μM iva (Vonk et al., 2020). FIS quantification was performed using Cell Profiler and Organoids Analyst Software (Hagemeijer et al., 2021), and the area under the curve (AUC) was calculated using GraphPad Prism 9.0.

### 2.4 Cell lines

Cell lines expressing wt-CFTR and p.Arg334Trp-CFTR were generated by lentiviral transduction of the human bronchial CFBE41o-cell line (which does not express any endogenous CFTR) after cloning of p.Arg334Trp-CFTR cDNA into lentiviral plasmids followed by site-directed mutagenesis as previously described (Canato et al., 2018; Awatade et al., 2019). CFBE cells expressing p.Phe508del-CFTR were a kind gift of Dr. Zsuzsa Bebok, United States (Bebok et al., 2005).

### 2.5 Western blot (WB) analysis

For CFTR protein detection, CFBE cells expressing p.Phe508del- or p.Arg334Trp-CFTR, respectively, were treated with 5 μM teza alone or combined with 3 μM elexa for 24 h, before being lysed and analysed by WB as described (Ramalho et al., 2021). Images were acquired using ChemiDoc XRS+ (BioRad, United States) imaging system and the intensity of the bands was quantified by Image lab 4.0 software (BioRad, United States).

### 2.6 Ussing chamber recordings

CFBE cells expressing p.Arg334Trp-CFTR were seeded on collagen IV (Sigma-Aldrich) -coated Snapwell filter inserts (Corning, United States) and treated as above with CFTR modulator drugs. CFTR function was assessed in Ussing Chamber as previously described (Awatade et al., 2019). The transepithelial voltage (Vte) and transepithelial resistance (Rte) were recorded and used to calculate the equivalent short-circuit current (Isc-eq) using Ohm’s law: Isc = Vte/Rte. Fsk was added apically followed by iva to cells pre-treated with DMSO or teza or elexa/teza for 24 h.

### 2.7 Statistical analyses

Statistical analyses were performed on GraphPad Prism 9.0 using ANOVA followed by a Dunnett’s test (*post hoc* test), with *p*-value ≤ 0.05 considered as significantly different.

## 3 Results

### 3.1 Assessment of p.Arg334Trp-CFTR function in intestinal organoids and the effect of CFTR modulators

To assess the response of the p.Arg334Trp CFTR variant to modulators we first used the forskolin-induced swelling (FIS) assay in pwCF-derived organoids as previously described (Vonk et al., 2020). The assay uses forskolin (fsk), a CFTR agonist, to induce organoid swelling. Fsk promotes the opening of the CFTR channel leading to ion and water uptake and consequent organoid swelling. In the absence of functional CFTR, there is no swelling, and the organoid keeps the same size. This assay has been validated as a robust *ex vivo* biomarker and as a good predictor of clinical benefit for CFTR modulators (Boj et al., 2017). Organoids were pre-incubated with the corrector teza alone or combined with elexa for 24 h and then, right before starting the FIS assay, they were acutely stimulated with fsk and iva. In total, we analysed rectal organoids from 10 individuals bearing the p.Arg334Trp variant (see Methods)—one homozygous, four heterozygous p.Arg334Trp/p.Phe508del, and five heterozygous for p.Arg334Trp with a zero-function variant (ZFvar) other than p.Phe508del (some authors call these “minimal function” variants, but this designation is misleading as it may be wrongly interpreted as “residual function”). In parallel, organoids from an individual homozygous for p.Phe508del were used as a positive control for response to the modulator drugs.

**FIGURE 1:**
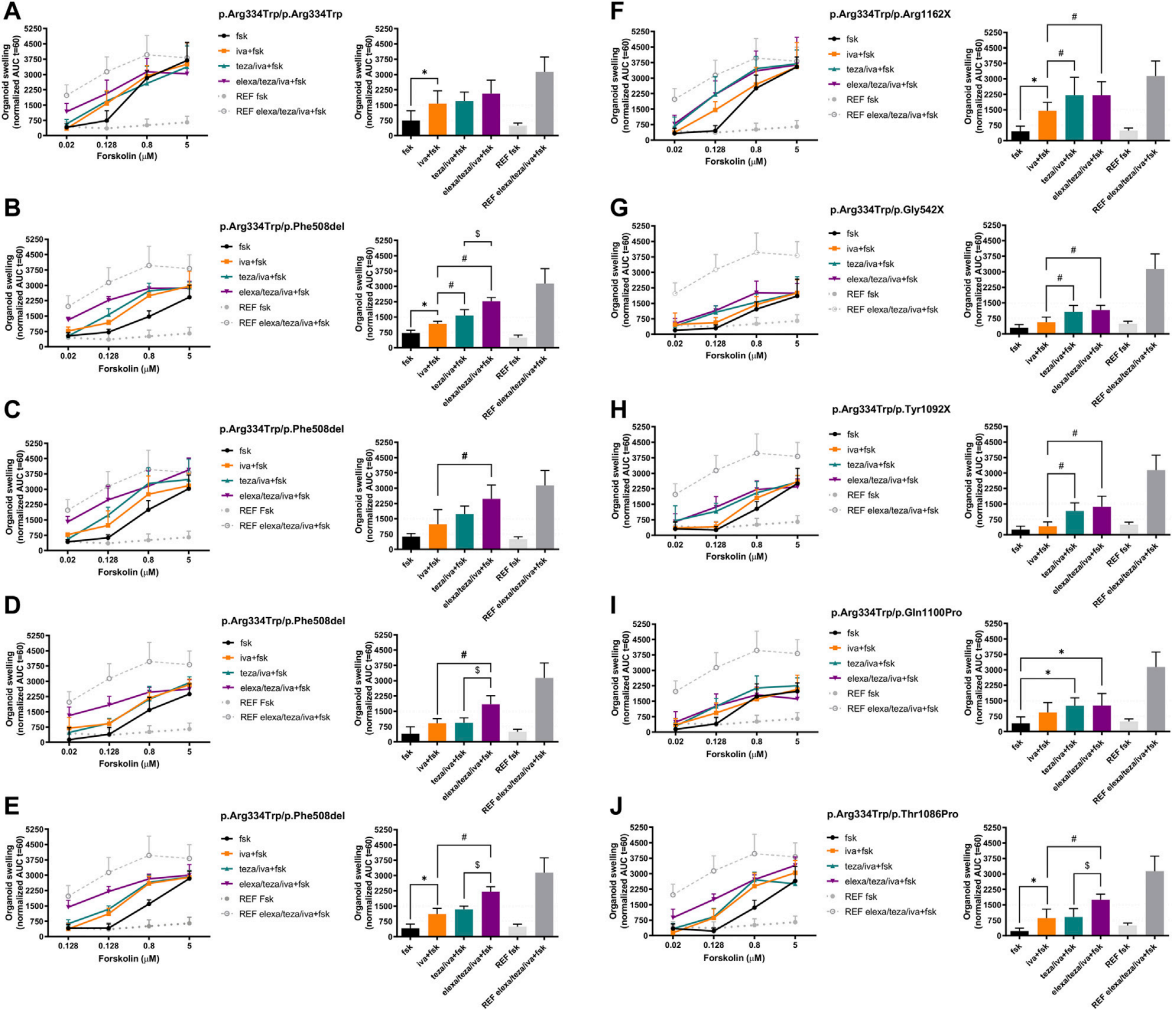
Forskolin-induced swelling (FIS) assay in intestinal organoids carrying p.Arg334Trp-CFTR. Organoid swelling is represented as area under the curve (AUC) of organoid surface area increase (baseline = 100%, t = 60 min). (Left) Organoids were tested at 0.02, 0.128, 0.8 and 5 μM of Forskolin (fsk) alone or in combination with only iva or after 24 h-incubation with teza or elexa/teza. (Right) Swelling (AUC) quantification of the organoids at t = 60 min for stimulation with 0.128 μM fsk alone or in combination with CFTR modulators. The p.Phe508del/p.Phe508del organoids treated with iva/teza/elexa were used as a reference for maximal CFTR rescue. Dashed green and red lines represent the established thresholds for high and medium potential clinical benefit for treatments, respectively. Data represent the mean ± SEM of 6–10 replicates per condition. Organoid genotypes are indicated above panels. Significance was considered for *p*-value ≤0.05 (ANOVA) and symbols represents significance vs.: *—stimulation with fsk alone; #—stimulation with iva + fsk; $—stimulation with teza/iva + fsk.

**FIGURE 2:**
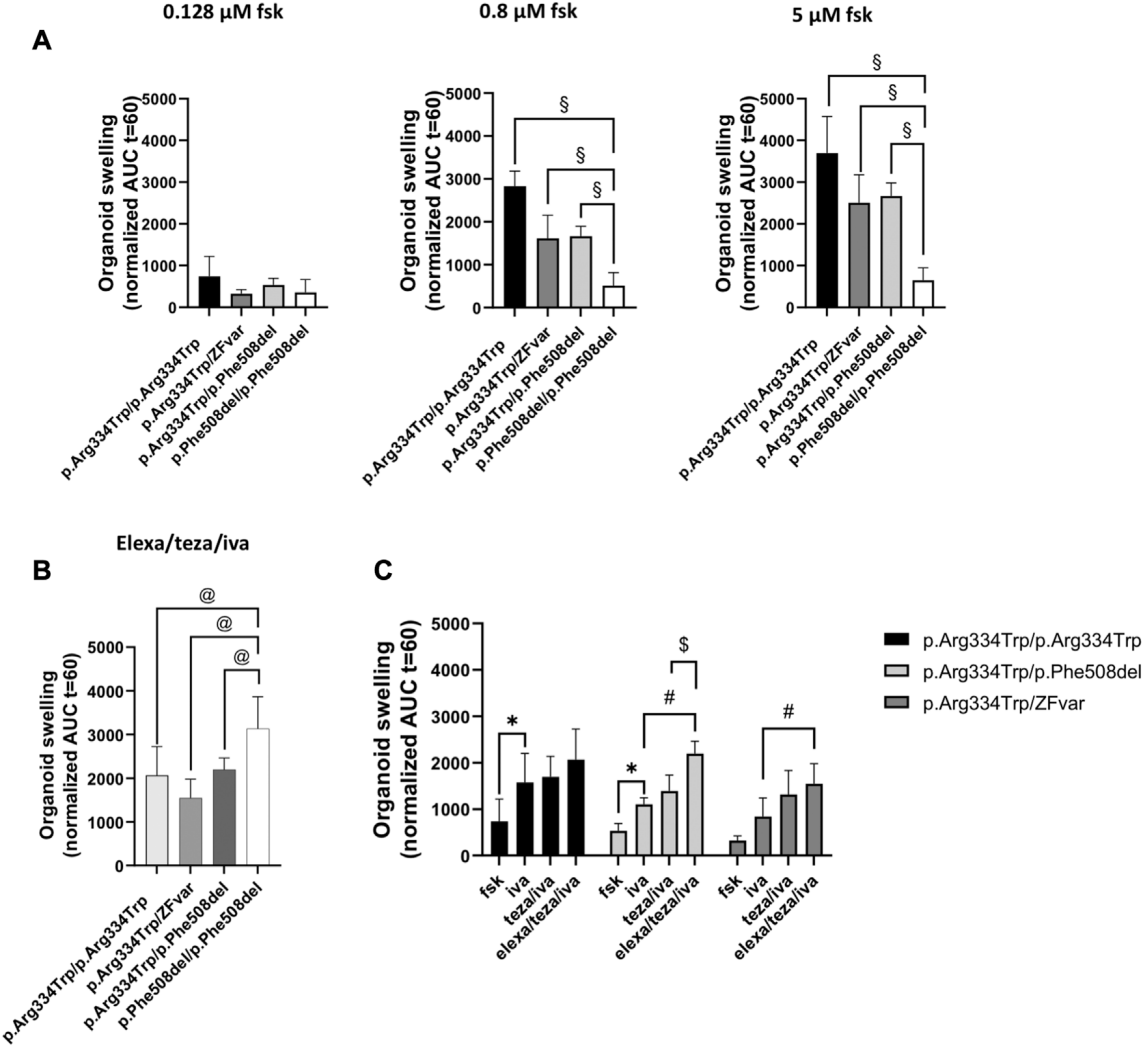
Comparison of the rescue of CFTR function by the three different modulators tested in intestinal organoids with p.Arg334Trp/p.Arg334Trp, p.Arg334Trp/ZFvar, and p.Arg334Trp/p.Phe508del genotypes. Organoid swelling is represented as area under the curve (AUC) of organoid surface area increase (baseline = 100%, t = 60 min) for stimulation with fsk alone or in combination with CFTR modulators. **(A)** Mean AUC of organoids carrying p.Arg334Trp/p.Arg334Trp, p.Arg334Trp/ZFvar, p.Arg334Trp/p.Phe508del at 0.128, 0.8 and 5 μM fsk alone **(B)** Mean AUC of organoids at 0.128 μM fsk treated with elexa/teza/iva. **(C)** Mean AUC of organoids carrying p.Arg334Trp/p.Arg334Trp, p.Arg334Trp/ZFvar and p.Arg334Trp/p.Phe508del at 0.128 μM Fsk treated with fsk alone or in combination with CFTR modulators. Data represent the mean ± SEM of 6–10 replicates per condition. Significance was considered for *p*-value ≤0.05 and symbols represents significance vs.: §—stimulation with fsk alone in p.Phe508del/p.Phe508del organoids (ANOVA); @- stimulation with elexa/teza/iva in p.Phe508del/p.Phe508del organoids (ANOVA); *—stimulation with fsk alone (ANOVA); #—stimulation with iva + fsk (ANOVA); $—stimulation with teza/iva + fsk (ANOVA).

Incubations with fsk alone show that organoids, either homozygous or heterozygous for the p.Arg334Trp variant, have residual CFTR function at fsk concentrations of 0.8 μM and higher when compared with p.Phe508del homozygous organoids (Figures 1A–J, left panel, black line vs. grey dotted line with full dots; Figure 2A, middle and right panels).

For the p.Arg334Trp-homozygous organoids, stimulation with potentiator iva alone resulted in significant organoid swelling when compared with the basal conditions (Figure 1A, right panel, orange vs. black bar). In these organoids, pre-treatment with teza or elexa/teza did not induced further significantly organoid swelling when compared to treatment with iva alone.

In organoids from two out of four pwCF with genotype p.Arg334Trp/p.Phe508del, treatment with potentiator iva alone at the 0.128 μM fsk concentration induced significant organoid swelling vs basal condition (Figures 1B, E, right panels, orange vs. black bar), but iva treatment was non-significant vs. baseline for the other two individuals (Figures 1C, D, right panels, orange vs. black bars). For organoids from these four pwCF, pre-treatment with teza only caused significant further swelling in organoids from one individual (Figure 1B, right panel, turquoise vs. orange bars), but not in those from the other 3 (Figures 1C–E, right panels, turquoise vs. orange bars). Non-etheless, a significant increase in swelling was always observed for organoids from these four individuals when stimulated with iva after pre-treatment with elexa/teza (Figures 1B–E, right panels, purple vs. orange bars).

Results for organoids from the five pwCF heterozygous for p.Arg334Trp and a non-p.Phe508del ZFvar, swelling after treatment with iva alone was only observed for organoids with the p.Arg1062X and p.Thr1086Pro variants (Figures 1F, J, right panels, orange vs. black bars, respectively). For these five organoids, pre-retreatment with teza only caused further significant swelling (vs. iva alone) when the second variant was p.Arg1162X, p.Gly542X, or p.Tyr1092X (Figures 1F–H, right panel, turquoise vs. orange bars), but not when it was p.Gln1100Pro, or p.Thr1086Pro (Figures 1I, J, right panels, turquoise vs. orange bars). However, for p.Arg334Trp/p.Gln1100Pro organoids teza/iva treatment caused significant swelling vs. fsk alone (Figure 1I, right panel, turquoise vs. black bars). The response of these organoids to elexa/teza/iva was similar to that observed for p.Arg334Trp/p.Phe508del organoids, i.e., in all five organoids significant swelling was observed vs. iva alone (Figures 1F–H, J, right panels, purple vs. orange bars) or vs. fsk (Figure 1I, right panel purple vs. black bars). However, the swelling observed for p.Arg334Trp/p.Phe508del and p.Arg334Trp/p.Arg334Trp organoids was higher by ∼30%, and by ∼25%, respectively from the swelling of organoids bearing p.Arg334Trp combined with non-p.Phe508del ZFvar, albeit these differences are not significant (Figure 2B). For organoids homozygous for p.Arg334Trp, treatment with potentiator iva was enough to rescue CFTR function, with no additional effect from the pre-treatment with correctors (teza and elexa) (Figure 2C, black bars). CFTR function was also rescued by iva alone in organoids heterozygous for p.Arg334Trp and p.Phe508del, however, elexa/teza/iva treatment resulted in further significant increase in organoid swelling (Figure 2C, light grey bars). For organoids heterozygous for p.Arg334Trp and a non-p.Phe508del ZFvar a significant increase in organoid swelling was only observed after treatment with elexa/teza/iva (Figure 2C, dark grey bars).

### 3.2 Impact of p.Arg334Trp on CFTR processing/traffic in cell lines and effect of correctors

**FIGURE 3:**
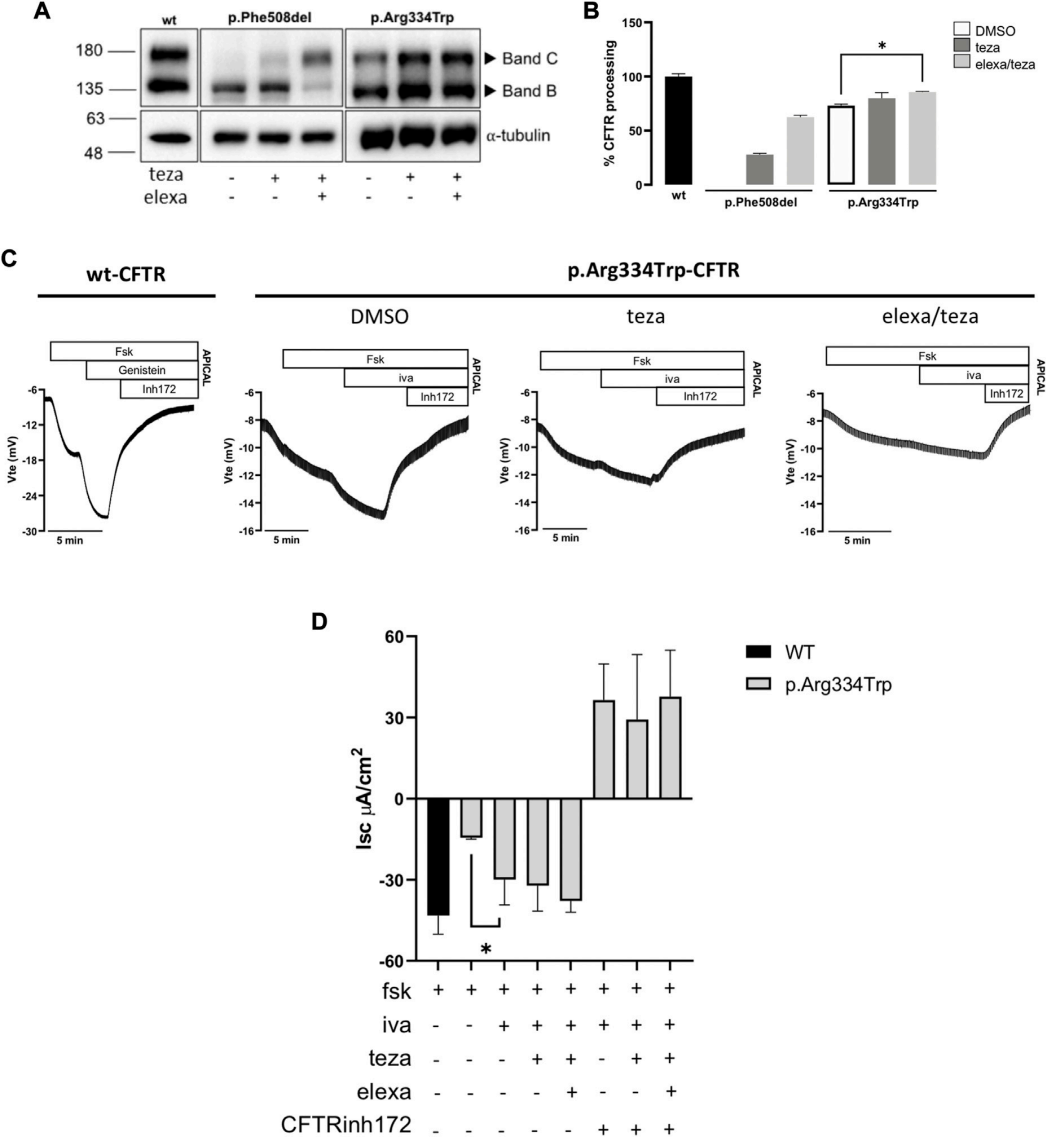
CFTR modulators effect on CFTR expression and function in CFBE cells expressing p.Arg334Trp-CFTR. **(A)** Western blot (WB) analysis of p.Arg334Trp-CFTR stably expressed in CFBE cells after 24 h treatment with 5 μM teza alone or combined with 3 μM elexa. Cells expressing F508del-CFTR were analysed as a control. As a loading control α-tubulin was used. **(B)** CFTR processing analysed as the percentage of band C to total CFTR and normalized to the efficiency of processing of wt-CFTR (shown as mean ± SEM of three replicates). Significance was considered for *p*-value ≤0.05 and * symbol represents significance vs. DMSO (ANOVA). **(C)** Original Ussing Chamber (short-circuit) recordings of CFTR-mediated chloride secretion measured as transepithelial voltage (V_te_). Fsk was added apically followed by iva to cells pre-treated with DMSO or 5 μM teza alone or combined with 3 μM elexa. DMSO was used as vehicle control. **(D)** Summary of equivalent short-circuit currents (I_sc-eq_) induced by fsk, iva + fsk, teza/iva + fsk and elexa/teza/iva + fsk. Values are represented as mean ± SEM (*n* = 4). Significance was considered for *p*-value ≤0.05 and * symbol represents significance vs. stimulation with fsk alone after DMSO (ANOVA).

To characterize the effect of p.Arg334Trp on CFTR traffic, function, and response to CFTR modulators, we produced a human bronchial epithelial (CFBE) cell line expressing this variant individually (see Methods). Cells were incubated for 24 h with 5 μM teza alone or combined with 3 μM elexa or treated only with DMSO 0.1% (v/v), as the vehicle control. In parallel, these compounds were also tested on p.Phe508del-CFTR expressing CFBE cells as a control. Total protein was collected, and WB was used to assess the maturation status as a proxy for PM traffic of p.Arg334Trp-CFTR. Results show that both mature and immature forms of p.Arg334Trp-CFTR were detected under control conditions (Figures 3A, B). In fact, p.Arg334Trp-CFTR processing efficiency (measured by the ratio of band C to total CFTR) was 73% of wt-CFTR. No significant effect of pre-treatment with teza alone was observed, while when teza was combined with elexa, a small albeit significant increase in p.Arg334Trp-CFTR processing (13% vs. DMSO) was observed.

### 3.3 Assessment of p.Arg334Trp-CFTR function in cell lines and effect of correctors

To evaluate the function of p.Arg334Trp-CFTR and its response to CFTR modulators, we assessed transepithelial ion transport in micro-Ussing chamber of CFBE cells stably expressing this variant. Residual CFTR-mediated Cl^−^ secretion was elicited by fsk addition, indicating that p.Arg334Trp-CFTR has indeed residual function which corresponds to ∼34% of wt-CFTR (Figures 3C, D). Similar to what we showed for the p.Arg334Trp-homozygous organoids, potentiation with iva alone was enough to rescue CFTR function. In fact, addition of iva alone further significantly increased the basal function by ∼36% vs. DMSO alone and to ∼69% of wt-CFTR (Figure 3D). Furthermore, consistently with the FIS data for the p.Arg334Trp-homozygous organoids, pre-treatment with correctors teza or elexa/teza did not further increase function of p.Arg334Trp-CFTR relative to iva alone (Figure 1A, right panel; Figure 3D).

## 4 Discussion

The rare p.Arg334Trp CFTR variant is described as CF-causing (CFTR, 2023) and it is not included in the list of variants eligible for treatment with the currently available CFTR modulators. In this study, we investigated the effect of CFTR modulators on p.Arg334Trp-CFTR in intestinal organoids derived from 10 pwCF having this mutation in homozygosity (1 individual) or in compound heterozygosity with p.Phe508del (4 individuals) or with a ZFvar: p.Arg1162X, p.Gly542X, p.Tyr1092X, p.Gln1100Pro, p.Thr1086Pro (1 individual each). Moreover, we characterized the p.Arg334Trp variant for CFTR protein traffic/processing and function when expressed alone in the CFBE cell line.

The p.Arg334Trp-CFTR is a class IV rare variant characterized by decreased CFTR conductance. In the CFTR2 database, there are 408 individuals registered who carry this variant. Clinically, p.Arg334Trp-CFTR is associated with atypical forms of CF, and individuals bearing this variant are likely to be pancreatic sufficient (CFTR, 2023).

To investigate CFTR function in intestinal organoids, we performed the FIS assay, which is fully CFTR-dependent (Boj et al., 2017). We observed that intestinal organoids either homozygous or heterozygous for p.Arg334Trp-CFTR show residual function at fsk concentrations of 0.8 μM and higher. These findings are in agreement with the results in the Ussing chamber for the p.Arg334Trp-CFTR variant stably expressed in CFBE cells which evidenced residual function (34% of wt-CFTR), which is consistent with defective conductance or lowered Po (Sheppard et al., 1993; De Boeck and Amaral, 2016; Han et al., 2018; Veit et al., 2020). Intestinal organoids carrying p.Arg334Trp/p.Phe508del variants showed lower residual CFTR function at higher concentrations of fsk stimulation when compared with p.Arg334Trp/p.Arg334Trp organoids. As p.Phe508del is a ZFvar, the observed residual function is most likely due to the presence of p.Arg334Trp.

Ivacaftor alone was able to significantly potentiate this residual CFTR function in p.Arg334Trp/p.Arg334Trp organoids and in 4/9 of heterozygous organoids for p.Arg334Trp. Our data show that organoids with the same p.Arg334Trp/p.Phe508del genotype evidenced variable responses to CFTR modulators (2 positive responses in four organoids), thus highlighting individual-specific differences in response to CFTR modulators. In the Ussing chamber, CFBE expressing p.Arg334Trp-CFTR also responded to iva stimulation by showing a further increase in function of 36% (reaching 69% of wt-CFTR). These data are also consistent with the FIS results in p.Arg334Trp-homozygous organoids that evidenced a 53% increase vs. basal swelling. It was previously shown that p.Arg334Trp-CFTR, when expressed in Fischer rat thyroid (FRT) cells, is normally processed and exhibits very low CFTR channel activity (Van Goor et al., 2014; Phuan et al., 2019), but with no further potentiation by iva. However, in CFBE cells, which are from human airway origin, a small but significant (Veit et al., 2020) increase has been observed upon iva treatment which is in line with our results. This discrepancy between FRT cells on the one hand and human bronchial epithelial cell line and pwCF-derived organoids on the other, highlight the superiority of the latter to predict responses to CFTR modulator drugs.

Western blot analysis for the CFBE cell line stably expressing p.Arg334Trp-CFTR detected both immature and mature forms of p.Arg334Trp-CFTR under control conditions, with a processing of 73% of wt-CFTR. Results also show that p.Arg334Trp-CFTR processing significantly increased by 13% after correction with the teza/elexa combination, but not with teza alone. Data from both FIS, in p.Arg334Trp-homozygous organoids, and Ussing chamber in CFBE cell expressing p.Arg334Trp-CFTR, show that pre-treatment with either teza or elexa/teza followed by stimulation with iva did not increase CFTR function when compared to iva treatment alone. This may appear as a discrepancy regarding the data on processing obtained by WB. However, it should be emphasized that cells analysed by WB were not subjected to iva. In fact, this compound has been shown to decrease the efficiency of correctors, which may explain the absence of an additional functional response (Cholon et al., 2014; Veit et al., 2014).

FIS data in organoids from 8/9 individuals heterozygous for p.Arg334Trp showed that the triple combination (elexa/teza/iva) induced a higher level of organoid swelling when compared to iva alone, suggesting additional rescue of CFTR by the elexa/teza combination. However, four of these eight organoids had p.Phe508del on the second allele, a variant known to respond to elexa/teza/iva. Indeed, the triple combination of elexa/teza/iva is approved for the treatment of individuals carrying only one copy of the p.Phe508del variant. It is important to mention that the effect of the triple combination in p.Arg334Trp/p.Phe508del was statistically lower, in all four subjects, when compared with the control p.Phe508del/p.Phe508del. These results suggest a low impact of the correctors teza and elexa on the rescue of p.Arg334Trp-CFTR.

Among the other four organoids which responded more to elexa/teza/iva than to iva alone were those with p.Arg334Trp-CFTR in combination with p.Thr1086Pro or with a PTC (premature termination codon) variant, namely,: p.Gly542X, p.Tyr1092X and p.Arg1162X. In fact, these three organoids also responded significantly more to teza/iva than to iva alone. As PTC variants are not likely to respond to modulators, the fact that response was only observed in the presence of correctors may suggest the need to increase maturation of p.Arg334Trp-CFTR for the potentiation effect of iva to be observed. In fact, we confirmed this increase in processing of p.Arg334Trp-CFTR by correctors in the cell line. Interestingly, the p.Arg334Trp/p.Arg1162X organoids showed a higher rescue of CFTR function, 48% and 38% more to elexa/teza/iva when compared with p.Arg334Trp/p.Gly542X and p.Arg334Trp/p.Tyr1092X, respectively. As the p.Arg1162X variant is closer to CFTR C-terminus, it could result in the production of small amounts of truncated CFTR partly rescuable by the modulators, as has been suggested for other non-sense variants such as W1282X (Haggie et al., 2017). Notwithstanding, these results may also result from a potential impact of modifier genes which contribute to the variation of CFTR rescue of those phenotypes (O’Neal and Knowles, 2018).

CFTR function rescue by CFTR modulators was also observed in intestinal organoids from individuals heterozygous for p.Arg334Trp-CFTR and p.Gln1100Pro or p.Thr1086Pro, respectively. The variant p.Gln1100Pro is very rare and characterized by defective CFTR protein folding (class II), which was shown to be rescued by the double combination of elexa/teza (Ramalho et al., 2021). The p.Thr1086Pro, is a missense variant and is not described in CFTR2 database. Both individuals carrying these variants are not eligible for the currently available CFTR modulator therapies. However, we show here that individuals having these variants combined with p.Arg334Trp are likely to have clinical benefit from CFTR modulator treatments, as the respective purple bars (Figures 1I,J, right panels) are above the dotted red line representing the established threshold for medium potential clinical benefit and very close (p.Arg334Trp/p.Gln1100Pro) or above (p.Arg334Trp/p.Thr1086Pro) the threshold for high potential clinical benefit (green dotted line). However, we do not know whether these variants in genotypes with other ZFvar will respond to CFTR modulators.

Taken together, our results provide evidence that p.Arg334Trp can be rescued by current CFTR modulators when in homo- or heterozygosity with a ZFvar. We can thus predict that individuals with this variant in at least one of their CFTR alleles will likely benefit from current CFTR modulator drugs (at least one orange, turquoise or purple bar in each right panel of (Figure 1), is above the red line and very close or above the green dotted line, representing the established thresholds for high and medium potential clinical benefit for treatments, respectively).

Although responses to teza/iva or to elexa/teza/iva in organoids with p.Arg334Trp in homozygosity or combined with p.Phe508del were usually higher than those in organoids with p.Arg334Trp combined with a non-p.Phe508del ZFvar, the differences were not found to be significant (Figure 2B). Moreover, as different responses were observed for the same genotype (p.Arg334Trp/p.Phe508del), as well as for a group of variants in the same class (p.Gly542X, p.Tyr1092X and p.Arg1162X), our data evidence the differences among individuals with the same genotype, thus highlighting the importance of personalized medicine in CF, which is of special relevance for pwCF carrying rare variants and thus unlikely to enter “classical” clinical trials.

Finally, as individuals with very rare variants are not eligible to participate in ‘classical’ clinical trials due to their low numbers (and geographic dispersion), we recommend that the precision/personalized approach adopted here should be taken into account for drug reimbursement policies by health insurance systems/national health services.

## Data Availability

The raw data supporting the conclusion of this article will be made available by the authors, without undue reservation.
